# Dimethylsulfoniopropionate concentration in coral reef invertebrates varies according to species assemblages

**DOI:** 10.1038/s41598-020-66290-5

**Published:** 2020-06-18

**Authors:** Isis Guibert, Flavien Bourdreux, Isabelle Bonnard, Xavier Pochon, Vaimiti Dubousquet, Phila Raharivelomanana, Véronique Berteaux-Lecellier, Gael Lecellier

**Affiliations:** 1 Swire Institute of Marine Science, The University of Hong Kong, Hong Kong S.A.R, China; 2 Sorbonne Université, UMR250/9220 ENTROPIE IRD-CNRS-UR-IFREMER-UNC, Promenade Roger-Laroque, Noumea cedex, New Caledonia, France; 3 USR3278 PSL CRIOBE CNRS-EPHE-UPVD, LabEx CORAIL, Papetoai, Moorea, French Polynesia; 4 Université de Paris-Saclay, UVSQ, 45 avenue des Etats-Unis, Versailles Cedex, France; 5 Institut Lavoisier de Versailles, UMR CNRS 8180, 45 avenue des Etats-Unis, Versailles Cedex, France; 6 USR3278 PSL CRIOBE CNRS-EPHE-UPVD, LabEx CORAIL, Université de Perpignan, 58 avenue Paul Alduy, 66860 Perpignan, France; 7 Coastal and Freshwater Group, Cawthron Institute, Private Bag 2, Nelson, 7042 New Zealand; 8 Institute of Marine Science, University of Auckland, Private Bag 349, Warkworth, 0941 New Zealand; 9 Délégation à la recherche, Government of French Polynesia BP 20981, 98713, Papeete, Tahiti French Polynesia; 10 UMR 241 EIO, Université de la Polynésie Française, BP 6570 Faaa, 98702 Faaa, Tahiti French Polynesia; 11 UMR250/9220 ENTROPIE IRD-CNRS-UR-IFREMER-UNC, Promenade Roger-Laroque, Noumea cedex, New Caledonia France

**Keywords:** Transcriptomics, Marine chemistry, Marine biology

## Abstract

Dimethylsulfoniopropionate (DMSP) is a key compound in the marine sulfur cycle, and is produced in large quantities in coral reefs. In addition to Symbiodiniaceae, corals and associated bacteria have recently been shown to play a role in DMSP metabolism. Numerous ecological studies have focused on DMSP concentrations in corals, which led to the hypothesis that increases in DMSP levels might be a general response to stress. Here we used multiple species assemblages of three common Indo-Pacific holobionts, the scleractinian corals *Pocillopora damicornis* and *Acropora cytherea*, and the giant clam *Tridacna maxima* and examined the DMSP concentrations associated with each species within different assemblages and thermal conditions. Results showed that the concentration of DMSP in *A. cytherea* and *T. maxima* is modulated according to the complexity of species assemblages. To determine the potential importance of symbiotic dinoflagellates in DMSP production, we then explored the relative abundance of Symbiodiniaceae clades in relation to DMSP levels using metabarcoding, and found no significant correlation between these factors. Finally, this study also revealed the existence of homologs involved in DMSP production in giant clams, suggesting for the first time that, like corals, they may also contribute to DMSP production. Taken together, our results demonstrated that corals and giant clams play important roles in the sulfur cycle. Because DMSP production varies in response to specific species-environment interactions, this study offers new perspectives for future global sulfur cycling research.

## Introduction

Coral reefs have been described as dimethylsulfoniopropionate (DMSP) hotspots^1,2^. This compound is an important metabolite that plays a central role in the marine sulfur cycle^3^. It is involved in numerous cellular and ecological processes. Among several known functions, DMSP possesses antioxidant properties^4,5^ as evidenced by cellular increase of DMSP under CO_2_ depletion^6^. Dimethylsulfoniopropionate has other protective physiological functions, notably serving as an osmolyte and cryoprotectant in marine algae^7,8^. Acting as a signalling molecule, DMSP is also involved in antiviral defense mechanisms and sulfide detoxification^9–11^. Numerous studies have recently drawn attention to variations of DMSP concentration in organisms subjected to environmental changes. Marine algae and/or coral studies have demonstrated that DMSP concentration changes with light intensity and salinity, as well as in response to oxidative stressors^6,12–14^. DMSP is therefore believed to be involved in organisms’ stress response, especially in coral species^14,15^. Dimethylsulfoniopropionate has also been extensively studied for its role in climate regulation^16^, because it can be converted into dimethylsulfide (DMS), a trace gas that is a source of reduced sulfur and which plays a role in cloud formation^17,18^. Consequently, precise methodologies such as nuclear magnetic resonance (NMR) spectroscopy have been developed for accurate detection and quantification of DMSP^16,19^.

Dimethylsulfoniopropionate is found only in certain terrestrial or marine organisms. It has been recorded in graminoids, rhodophytes, mussels and also in benthic flatworms^20–23^. However, only a few species are able to produce DMSP, and the majority of organisms are believed to accumulate this molecule through their diets or phototrophic symbionts^24^. To date, pathways of DMSP biosynthesis have been characterized in higher plants^22,25^, marine algae such as Symbiodiniaceae^26,27^ and more recently in corals, with methionine as a common precursor^15^. Higher plants and marine algae use two different DMSP biosynthetic pathways, while corals harbor an algal-like pathway^15,26^. Similarly to marine algae, coral species such as *Acropora* sp. likely encode the enzymatic machinery required for biosynthesis of DMSP (specific aminotransferase, reductase, methyltransferase and decarboxylase enzymes)^3,15^. Even though the specific biosynthesis pathway has not yet been studied in other marine organisms, DMSP has been found in other coral taxa (e.g. Pocilloporidae, Poritidae) and sessile organisms such as giant clams and anemones^20,28^. The concentrations of DMSP were higher in corals (*Acropora* sp., *Heliopora* sp., *Pavona* sp.) and giant clams (*Tridacna maxima* and *Tridacna squamosa*) than in other animals^20,28^. Dinoflagellates of the family Symbiodiniaceae account for most of the DMSP production in coral reefs^24,29^. This family is currently classified into nine divergent lineages, hereafter referred to as clades A to I^30^, of which seven have recently been elevated to genus level^31^, with clades A-D commonly found in symbiosis with scleractinian corals and giant clams^32^. Symbiodiniaceae can possess a DMSP-lyase activity leading to the conversion of DMSP into DMS that differs among clades^33^. Thus, the presence or proportion of different Symbiodiniaceae clades within a host may influence the variation of DMSP concentration often observed between coral species^34^. Additionally, the differences observed in coral studies could also be the result of other symbionts hosted by corals. Certain bacteria possess crucial genes for DMSP degradation such as homologs of the *dmdA* gene that are involved in the demethylation pathway or of the *dddD* gene known to be involved in the degradation of DMSP into DMS^35–38^. These homologs have been found in Proteobacteria that are common in coral species. Additionally, the occurrence of bacteria involved in DMSP production in coral is possible^39^, further highlighting the complexity of DMSP-cycling processes in benthic holobionts. Most of these bacteria have been recently described in microbiomes of giant clams^40^. Further, DMSP production in heterotrophic bacteria has also been recently highlighted^41^.

It is well known that coral symbionts such as dinoflagellates (Symbiodiniaceae) and bacteria vary according to environmental conditions^42–46^, and are therefore assumed to play a key role in their host fitness^47^. In addition to symbionts, interaction between holobionts could also impact the host fitness. While it is well known that marine sessile organisms interact, either directly or indirectly^48–50^, only a few studies have underlined the role of benthic species assemblages on coral reef dynamics^51,52^, as well as on giant clams’ fitness^40^. To better understand how benthic species assemblages could influence their respective fitness, we artificially combined three coral reef builders, including two coral species (*Pocillopora damicornis*; Linneaus, 1758 and *Acropora cytherea*; Dana, 1846) and one giant clam species (*Tridacna maxima*; Röding, 1798), and measured DMSP concentration in each species using NMR spectroscopy. Metabarcoding was used to characterize the Symbiodiniaceae composition within the three studied species and determine the putative role of Symbiodiniaceae in DMSP production. We also examined whether giant clams contribute to DMSP production by screening our previously described transcriptomes of *T. maxima*^53^ for enzymes involved in the DMSP biosynthesis. Finally, because DMSP is a stress biomarker, the experiments were performed either at lagoon temperature or under thermal stress.

## Material and Methods

### Coral and giant clam collection and experimental design

The coral and giant clam samples as well as the experimental design used in this study were described previously^52^. Briefly, coral species of *A. cytherea* and *P. damicornis* were collected in Moorea lagoon, French Polynesia (17°30′S, 149°50′W, Linareva fringing reef)^54^. For each species, 4 colonies were sampled and nubbinized into 45 small fragments. Giant clams of the species *T. maxima* were purchased from a French Polynesian nursery on Reao Island (18°28′S, 136°25′W; N°Tahiti: 139 519). Corals nubbins and giant clams were reared in a common garden. A CITES permit was obtained to allow specimen exports (CITES – FR1698700087 – E).

Experiments were conducted in open-circuit aquariums (20 L/h), using distinct assemblages of either one species *P. damicornis* (P, n = 4 aquariums) or *T. maxima* (T, n = 4 aquariums), two species *P. damicornis* + *A. cytherea* (PA, n = 4 aquariums) or *A. cytherea* + *T. maxima* (AT, n = 4 aquariums), or three species *P. damicornis* + *A. cytherea* + *T. maxima* (PAT, n = 4 aquariums; Figure S1a). Three nubbins per coral colony (n = 3 nubbins x 4 colonies per species) and 12 giant clams were used when required in an assemblage. Coral fragments (5–8 cm in height and 2 cm in diameter) and giant clams (4–8 cm in size) were spaced 5 to 10 cm apart to avoid contact. Assemblages were either under thermal stress (32 °C, S, n = 10 aquariums) or at lagoon temperature (27 °C, L, n = 10 aquariums). After 12 days of acclimation at 27 °C (time 0), half of the aquariums (n = 10) were placed under thermal stress by increasing the temperature by 1 °C per day until it reached 32 °C on day 17 (Fig. S1b). At each sampling time (day 12 and 17), half of the sampled giant clam mantles (4 cm^2^) and 80% of the sampled coral fragments were immediately snap-frozen in liquid nitrogen and stored at −80 °C until further analysis. The remaining portions of the sampled giant clams and corals were stored in 70% ethanol for DNA analysis. Seawater temperature data were recorded every 10 minutes with Temperature/Light Data Loggers (P/N U22–001, Onset, Bourne, Massachusetts; or Ruskin, Ottawa, Canada; Fig. S1b). Seawater temperature was controlled with the Biotherm pro system (Hobby, Stukenbrock, Germany). Health status checks on the coral nubbins were carried out using the standardized coral coloration scale as a proxy of symbiont density and chlorophyll a content variation (Coral Health Chart, www.CoralWatch.org)^55,56^, and through daily visual observation for giant clams.

### Metabolite extraction for dimethylsulfoniopropionate quantification

Coral samples were lyophilized overnight, and 1 to 1.5 g of powder was obtained for extraction. The extraction protocol was adapted from Tapiolas and collaborators^19^. In total, 51 *A. cytherea* and 54 *P. damicornis* samples were extracted with 3 mL of HPLC-grade methanol (CH_3_OH). After 5 minutes of sonication (35 kHz Transsonic 950/H, Elma, Germany) at room temperature, the extracts were shaken for 3 hours. A second extraction with an additional 2 mL of CH_3_OH was performed using 5 minutes of sonication and 5 minutes of shaking. The two extracts were then pooled and dried using a vacuum centrifuge drier (Genevac EC-2 plus, Genevac, UK) before being stored at −20 °C until NMR analysis. Samples were lyophilized overnight and weighed to measure the quantity of extracts.

Giant clam samples (n = 42) were lyophilized overnight and 0.1 to 0.5 g of powder was obtained for extraction. Protocol extraction was adapted from Mohamadi^57^. Solid-liquid extractions were performed using 2 mL of H_2_0 and 0.5 mL of HPLC-grade methanol (CH_3_OH) prior to vortexing. After adding 1.5 mL of CH_3_OH and 2 mL of dichloromethane (CH_2_Cl_2_), the solutions were mixed using a vortex and sonicated for 10 minutes. The solid-liquid extraction was carried out three times and the three extracts were pooled and centrifuged at 2050 g for 20 min in order to separate the polar and apolar phases. The apolar extracts were pooled as per the polar extracts and lyophilized. In this study, only the hydroalcoholic (polar) extracts of the giant clam samples were analyzed.

All extracts were resolubilized in 500 μL of deuterium oxide (D_2_O), vortex mixed and transferred into a 5 mm NMR tube, then analyzed immediately by ^1^H-NMR spectroscopy.

### Nuclear magnetic resonance data acquisition and dimethylsulfoniopropionate quantification

All ^1^H spectra were recorded on a Bruker Avance 1 spectrometer (Bruker, Germany) at 300 MHz in the same conditions with 128 transients; chemical shifts were reported in ppm from tetramethylsilane (TMS). Absolute Area was determined by NMR analysis on the topspin 2.1 Bruker software.

Two external calibration curves of DMSP were performed under the same conditions as the samples with 128 transients (Figure S1c,d). Integration zone A (3.41 ppm to 3.34 ppm) was used for *A. cytherea* and *P. damicornis* samples because there were no overlapping signals. Integration zone B was used for *T. maxima* samples because of overlapping signals on zone A and no overlapping signal on zone B (Fig. S1e–h). The concentrations of DMSP calculated with the calibration curve (in mg per mL) were normalized with the mass ratio corresponding to the dry weight of the extract. Analysis of variance (ANOVA) and *ad hoc* pairwise comparisons (Tukey test) were performed using R software^58^.

### DNA extraction; PCR amplification and sequencing of Symbiodiniaceae communities

Coral DNA extractions (n = 2 to 9 per assemblage) were performed following a Cethyl Trimethylammonium Bromide (CTAB) based protocol^59^. Symbiodiniaceae diversity was studied using the internal transcribed spacer 2 (ITS2) marker. The ITS2 region of the nuclear ribosomal array was amplified using the forward primer “its-Dino” (5′-GTGAATTGCAGAACTCCGTG-3′) and the reverse primer “ITS2-Rev2” (5′-GCCTCCGCTTACTTATATGCT-3′)^60,61^. Illumina^TM^ overhang adaptors were included in the primers as described in Kozich *et al*.^62^. PCR amplification and sequencing were performed as described in Guibert *et al*.^52^.

Symbiodiniaceae composition analysis of *A. cytherea* and *P. damicornis* samples was adapted from the Arif *et al*. (2014) pipeline in mothur v1.39^63^. An ITS2 database^64^ was used to annotate the sequences via Basic Local Alignment Search Tool (BLASTN). The relative abundance of Symbiodiniaceae subclades was represented using R (Reshape2 and ggplot2 packages).

Symbiodiniaceae composition analysis of *T. maxima* samples was processed using the same methodology as described in Guibert *et al*.^40^.

### Identification of candidate genes

A previous predicted peptide data set of *T. maxima* metatranscriptome^53^ was blasted against a custom database at a threshold of 10^−3^. This database contained molluscan and cnidarian sequences extracted from the Protein database of the National Center for Biotechnology Information (NCBI) filtered with the DMSP enzyme names or EC numbers and from the DMSP candidate genes previously identified in *Acropora millepora*^15^ (Table S2, sheet 2). The selected sequences were blasted against the whole NCBI Protein database and any sequence with a top hit with a plant, algae or bacterial taxonomy was considered a false positive and removed (Table S2, sheet 1).

A differential gene expression analysis was then performed from previous RNAseq results of *T. maxima* under thermal stress at 32 °C for 5 days (Bioproject accession number: PRJNA309928). Transcript quantification of the above selected sequences was performed with RNA-Seq by Expectation Maximization (RSEM)^65^ across 14 samples of the Bioproject (8 biological samples: B0Tx and BT0 as the control group and 6 BTx biological samples as the Heating group^53^). The differential expression between the 2 groups was performed with the edgeRun package^66^ with 50,000 iterations.

## Results

### Analyses of dimethylsulfoniopropionate concentrations according to thermal stress, species and assemblages

Dimethylsulfoniopropionate concentration was evaluated by ^1^H-NMR spectroscopy and standardized with the mass ratio in the three species: *P. damicornis*, *A. cytherea* and *T. maxima* (Table S3). An ANOVA test (Table 1) revealed significant differences among assemblages (p = 1.01e-11) or species (p < 2e-16) with a significant interaction among them (p = 3.90e-08). No significant differences between assemblages and temperatures were found. Thus, the DMSP data were pooled by assemblages for each species.

**Table 1:** Results of ANOVA test on DMSP concentration (mass ratio) by assemblage, species and temperature. Df: degrees of freedom; SumSq: Sum of squares; Mean Sq: mean sum of squares, F value: F statistic.

	Df	Sum Sq	Mean Sq	F value	P value
Assemblage	5	184.3	36.86	15.274	1.01e-11***
Temperature	1	2.7	2.67	1.107	0.295
Species	2	339.5	169.75	70.343	<2e-16***
Assemblage: Temperature	4	11.3	2.81	1.166	0.329
Assemblage: Species	2	94.6	47.28	19.594	3.90e-08***
Temperature: Species	2	4.1	2.05	0.848	0.431
Assemblage: Temperature: Species	2	0.3	0.14	0.060	0.942
Residuals	126	304.1	2.41		

**Figure 1:**
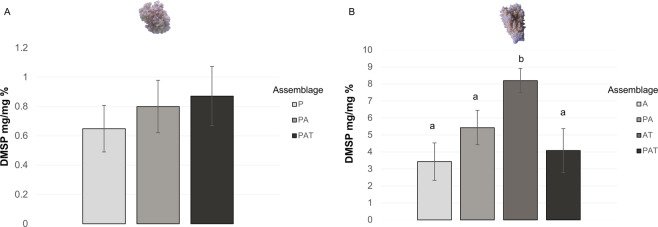
Concentration of dimethylsulfoniopropionate (Mass ratio: mg/mg %) of *Pocillopora damicornis* (**A**) and *Acropora cytherea* (**B**) by assemblage. Assemblages: P: *P. damicornis* (nA = 25); A: *A. cytherea* (nB = 12); PA: *P. damicornis* and *A. cytherea* (nA = 16, nB = 16); AT: *A. cytherea* and *T. maxima* (nB = 8); PAT: *P. damicornis*, *A. cytherea* and *T. maxima* (nA = 13, nB = 15). Letters in lower case indicate significant differences between means (Tukey, p < 0.05). Photographs: Isis Guibert.

In corals, the concentrations of DMSP were from 5 to 10-fold lower in *P. damicornis* than in *A. cytherea* (Tukey test, p < 0.001), with means ranging between 0.65 and 0.87% (% = mg DMSP/100 mg extract) for *P. damicornis* and between 3.43 and 8.20% for *A. cytherea* (Fig. 1). In *P. damicornis*, while mean DMSP concentrations increased with the increasing complexity of assemblages, no significant differences were found (Fig. 1A). However, in *A. cytherea*, the DMSP concentration increased for the two-species assemblages (PA and AT) but decreased in PAT assemblages. A significant difference was found between AT and the other assemblages (A, p < 0.0001; PA, p < 0.05; PAT, p < 0.001; Fig. 1B).

**Figure 2:**
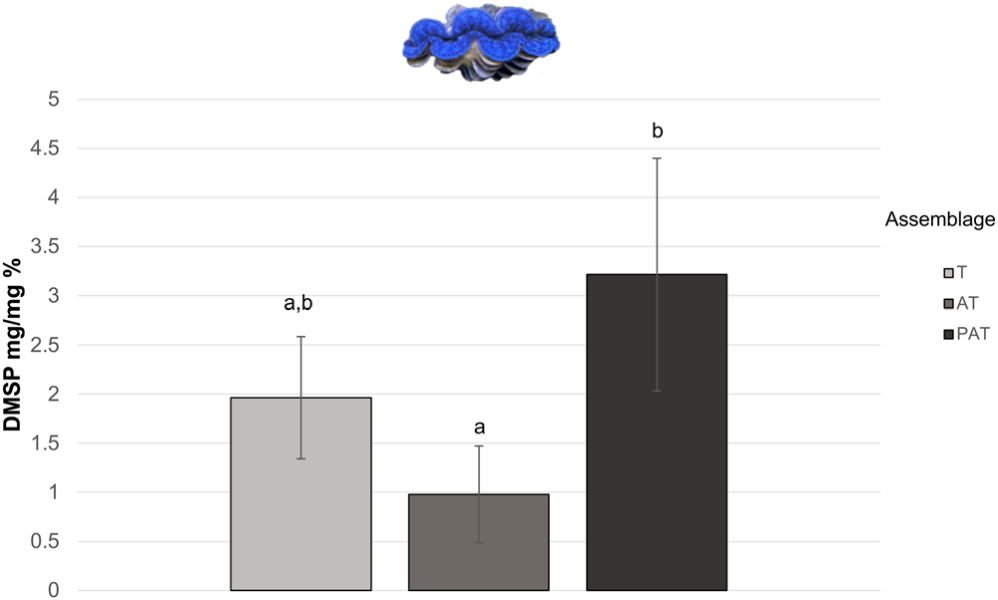
Concentration of dimethylsulfoniopropionate (Mass ratio: mg/mg %) of *Tridacna maxima* by assemblage. Assemblages: T: *T. maxima* (n = 19); AT: *A. cytherea* and *T. maxima* (n = 9); PAT: *P. damicornis*, *A. cytherea* and *T. maxima* (n = 14). Letters in lower case indicate significant differences between means (Tukey, p < 0,05). Photograph: Isis Guibert.

Interestingly, in *T. maxima*, mean DMSP concentrations decreased when exclusively associated with *A. cytherea* (from 1.97% to 0.98%) but increased in the PAT assemblage (3.22%, Fig. 2). *Tridacna maxima* exhibited a significantly higher DMSP concentration in the PAT assemblages than in the AT assemblages (p < 0.5).

### Symbiodiniaceae composition by assemblage

**Figure 3:**
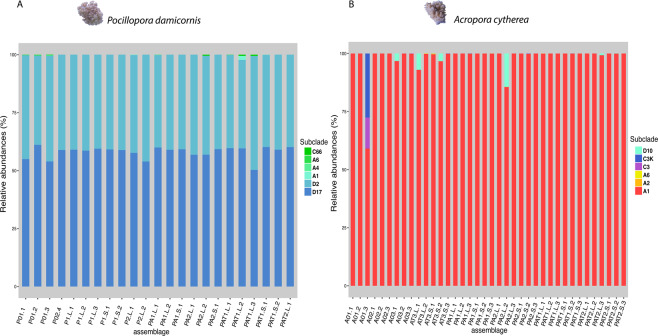
Relative abundance of Symbiodiniaceae subclades in *Pocillopora damicornis* (**A**) and *Acropora cytherea* (**B)**. All samples were collected at day 17 except those with a 0 in their name that were collected at day 12. PAT: *P. damicornis*, *A. cytherea* and *T. maxima*; AT: *A. cytherea* and *T. maxima*; A: *A. cytherea*; P: *P. damicornis*; 0: time 0 - day 12; (1–3): number of the experiment; L: lagoon temperature; S: thermal stress; (1–4): number of sample. Photograph: Isis Guibert.

The relative abundances of Symbiodiniaceae genotypes associated with the different assemblages and thermal stress conditions were measured at the clade and subclade levels (Fig. 3 and Table S4). Among the three Symbiodiniaceae clades (A, C and D) detected in the samples, clade D was the dominant lineage in *P. damicornis* nubbins (> 90%; Fig. 3a). Two D subclades were systematically detected in all nubbins. Subclade D17 represented more than half of the relative abundance (58–60%) and subclade D2 ranged from 39 to 44%. Clades A and C were only detected as background clades with subclades A1, A4, A6 and C66 sporadically present in the nubbins, either alone or associated, regardless of temperature levels and/or assemblages.

Three clades (A, C and D) were also detected in *A. cytherea* (Fig. 3b), although clade A largely dominated in all nubbins (n = 38), 24 of which harbored only this clade. Subclade A1 was dominant in the Symbiodiniaceae composition of all *A. cytherea* nubbins, with the presence of A2 subclade only once, and A6, 5 times, with both subclades occurring at a background level (<1%). Clade C was detected in 9 nubbins at the background level (<0.2%) except for one nubbin (A01.3 composed of 59% A and 41% C). Two C subclades, C3 and C3K were found concomitantly (in 4 nubbins) or separately (in 2 and 3 nubbins, respectively). Finally, clade D (subclade D10) was found in 5 nubbins, two of which contained D10 above the background level (7% and 14%). No significant correlation between Symbiodiniaceae clade/subclade and host assemblages or temperature was found. Moreover, no variation of nubbin color, was recorded according to temperature levels and/or assemblages meaning that there was no drastic variation in Symbiodiniaceae densities.

### Identification of candidate genes for dimethylsulfoniopropionate biosynthesis in Tridacna maxima

**Figure 4:**
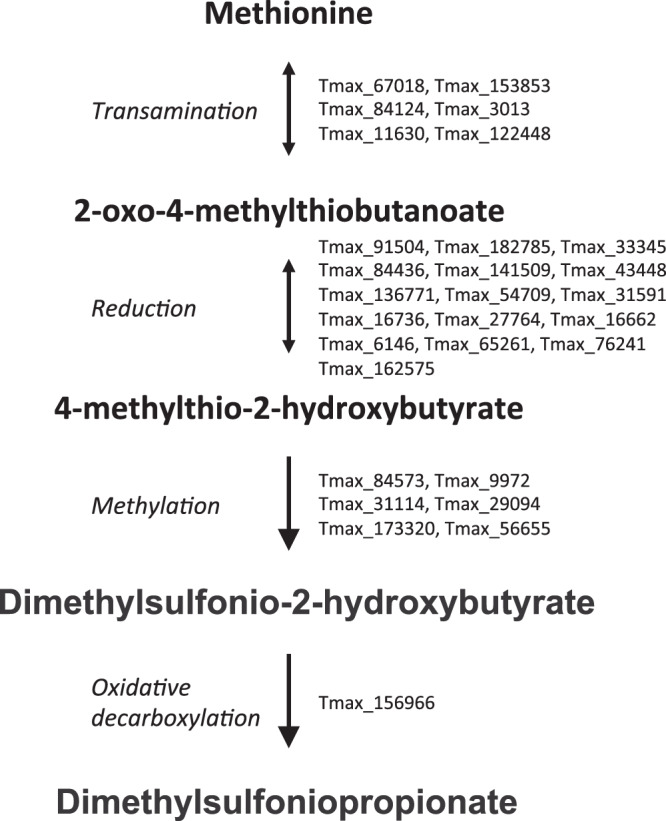
Putative pathway of dimethylsulfoniopropionate biosynthesis in *Tridacna maxima*. ID numbers of *T. maxima* peptides are indicated for each step.

The blasting of the predicted peptide *T. maxima* dataset against a DMSP enzyme custom database allowed the selection of 475 predicted peptides potentially involved in pathways of DMSP biosynthesis (Table S2 and S5, sheet 2). After functional clustering, all identified peptides with potential roles in DMSP synthesis were found to exhibit at least 1 homolog per step. Among them, 69 were significantly (False Discovery Rate <0.05) up-regulated during a thermal stress event (Table S5, sheet 1). More precisely, 29 peptides were identified as homologs to those of the coral pathway: 6 homologs of AT1 and AT5 for transamination step, 16 for the reduction step, 6 homologs of REDOX2,3,4,6 and 10, for the methylation step of METHYL 1 and 2, and one homolog of DECARB3 for the decarboxylation step (Fig. 4).

## Discussion

### Dimethylsulfoniopropionate concentrations differ according to species and assemblage

Dimethylsulfoniopropionate concentrations varied among the three invertebrate species analyzed. In *A. cytherea*, DMSP concentrations were 5 to 10-fold higher than in *P. damicornis*. A previous study performed on Pocilloporidae and Acroporidae, measured DMSP concentrations of, respectively, 0.333 nmol/mm^2^ and 2.473 nmol/mm^2^ in *P. damicornis* and *A. millepora*^19^, which is consistent with our results. Also, similar DMSP concentrations were found in three different species from the Pocilloporidae family, while they were highly variable in Acroporidae^19^. As we used different NMR quantification approaches for corals and giant clams, we were not able to directly compare concentrations between them. Nevertheless, beyond differences between species, our results revealed that the DMSP concentrations might vary in a species according to its close environment, namely interspecies assemblages.

In corals, increases in DMSP have been associated with direct sunlight, thermal stress, air exposure or oxidative stressors^3,5,14^. Measurements of DMSP concentration in *Acropora* species (*A. aspera*, *A. tenuis* and *A. millepora*) after 4^67^ and 5^3^ days at 31 °C revealed an increase of DMSP concentration in bleached corals. Here we show that after 2 days at >31 °C the DMSP production, in still unbleached *A. Cytherea*, *P. damicornis* and *T. maxima*, is not sufficiently enhanced to detect a significant difference in DMSP concentrations between stressed and unstressed corals and giant clams. This indicates that at least three days of temperature stress and/or the concurrent loss/gain of a certain quantity of Symbiodiniaceae/bacteria might be required for enhancing DMSP production. However, significant variations of DMSP concentrations were observed among the three species. These variations occurred in different environmental contexts. For *A. cytherea*, DMSP concentration increased significantly in AT assemblages but not in PA and PAT assemblages, whereas for *T. maxima*, it increased in PAT assemblages but decreased in association with *A. cytherea*. These previous results suggest that DMSP concentration in coral reef holobionts is dependent on neighboring species, possibly acting as a stressor^14^ or an activator of the sulfur metabolism. For *P. damicornis*, the association with *A. cytherea* alone or *with T. maxima* does not lead to a highly significant increase in DMSP. For *A. cytherea*, an increase of DMSP is observed in the AT assemblage. However, when *P. damicornis* is also present in the assemblage (PA or PAT), this effect disappeared. Therefore, the presence of *P. damicornis* prevents the clams effect’, highlighting possible yet uncharacterized interspecies communication responses. It is also known that, depending on the species, the variation in DMSP is not always observed under the same environmental factor. For example, a decrease in salinity leads to a decrease of DMSP in *A. millepora*, but such an effect had not previously been observed in *Stylophora pistillata* and *P. damicornis*^68^. In the present study, contrary to *A. cytherea*, the DMSP concentration in giant clams was increased in PAT assemblages rather than in AT assemblages. For giant clams, the presence of the two coral species seems to have a stronger effect than the sole presence of *A. cytherea*. In the case of DMSP as an indicator of stress, we have previously shown that giant clam mortality occurred in presence of *A. cytherea*, in both AT and PAT assemblages^40^. However, giant clam DMSP increases occurred only in PAT assemblages and we did not find any correlation between health status and DMSP levels in giant clams. Thus, the “health status” (i.e. declining clams) does not appear to systematically lead to a significant change in DMSP level. Taken together, our results suggest that DMSP concentration in the holobionts is influenced by their neighboring species, modifying the metabolism of the sulfur pathway.

### Dimethylsulfoniopropionate production

The variations in DMSP concentrations among invertebrates harboring Symbiodiniaceae have been essentially attributed to the types and densities of their symbiotic dinoflagellates^20^. Significant DMSP variation also exists between Symbiodiniaceae clades^5,20,33,67^. Consequently, the types and densities of Symbiodiniaceae possibly impact the DMSP concentrations in holobionts, by translocating DMSP to their hosts^69,70^. Nevertheless, DMSP concentrations and the types of clades in our samples were not correlated. While the DMSP concentration of *A. cytherea* was high in AT assemblages and low in PAT assemblages, the relative abundance of clade A and D were similar in both assemblages. If Symbiodiniaceae are indeed responsible for the DMSP concentrations, we have to assume that their density should be higher in *A. cytherea* nubbins in AT than in other assemblages. Moreover, as DMSP concentrations in *P. damicornis* nubbins are between 5 and 10-fold lower than in *A. cytherea* nubbins, and since clade D has been described as a lower DMSP producer than clade C^67^, we can also hypothesize that clade D is a lower DMSP producer than clade A, a permanent clade in *A. cytherea*. Another non-exclusive alternative hypothesis for explaining the difference in DMSP production between *A. cytherea* and *P. damicornis* relies on a difference in Symbiodiniaceae densities between these two coral species, as observed in our previous work, *A. cytherea* harboring more Symbiodiniaceae than *P. damicornis* in Moorea^59^. Regarding giant clams, as for corals, Symbiodiniaceae types are not sufficient to account for DMSP concentrations variations between assemblages. At the individual level, giant clams in the same assemblage, with either 99% of subclade C66 or 99% of subclade A6 did not exhibit any difference in DMSP concentration^40^. Thus, even if the Symbiodiniaceae are well known DMSP producers, our results suggested that Symbiodiniaceae genotypes did not account for the DMSP variations observed in our study.

**Figure 5:**
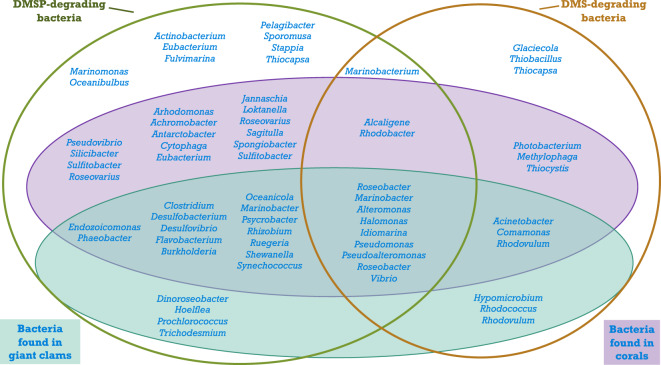
Marine bacterial genera degrading DMSP or DMS in the water column, associated or not with corals and/or giant clams. DMSP-degrading (green circle) and DMS-degrading (brown circle) found in the water column, bacterial genera found in corals (blue oval) and in giant clams (green oval) (adapted from^37,40^; and further developed from^71–77^).

In addition to Symbiodiniaceae, corals and giant clams harbor numerous other associated organisms, including various families of bacteria. Raina and collaborators^37^ reported that more than 65% of the bacteria genera playing a role in DMSP/DMS metabolism can be found in association with coral species. The bacterial communities associated within the giant clams of this experiment have already been characterized^40^. By overlapping the data from both studies, we demonstrated that several bacterial genera involved in the degradation of DMSP or DMS in corals were also associated with *T. maxima* (Fig. 5). This suggests that the microbial communities of giant clams are also structured by the presence of DMSP and could indicate that bacteria influence the composition and variation in DMSP concentration between assemblages. However, as for Symbiodiniaceae, no link was found between bacterial composition and assemblages in *T. maxima*. Altogether our results suggested that the variation in DMSP concentration observed here did not result from Symbiodiniaceae or bacterial genera present in the sample, but might result from differences in their relative densities.

Recent studies have highlighted the existence of DMSP biosynthesis pathways in animals, and in corals notably^3,15^. The increase in DMSP concentration measured in some assemblages might also be due to an enhancement in DMSP biosynthesis by the hosts, including giant clams. Of relevance, we showed for the first time that, similarly to corals, the giant clam *T. maxima* possesses algal gene homologs putatively involved in DMSP biosynthesis pathways. Candidate genes were not only homologous to DMSP alga-like pathway genes (top blast hit), but were also overexpressed during a thermal stress event. As at least one candidate gene for each step of the alga-like pathway has been found, we assume that the whole pathway is present in *T. maxima*. Future studies on complete holobiont communities and gene expression by assemblage will be required to decipher the involvement of each holobiont partner in DMSP biosynthesis.

This study explored the influence of interspecies assemblages on DMSP concentrations in corals and giant clams. We showed that DMSP concentration is higher in *A. cytherea* when associated with *T. maxima* and higher in *T. maxima* when they are in a three species assemblage. Because the balance between Symbiodiniaceae genera was maintained regardless of host assemblages, our results also show that microbial communities cannot solely account for the DMSP differences observed within and among assemblages. Further studies are required to decipher if symbiont densities and/or the host alga-like DMSP biosynthesis pathway contribute to the differences in DMSP production between assemblages.

## Supplementary information

Supplementary data S1.

Supplementary data S2.

Supplementary data S3.

Supplementary data S4.

Supplementary data S5.

## Data Availability

Supplementary data for this article can be found at Aquatic Sciences Website. All data are available upon request.
