# Supplementary material for: Dimethylsulfoniopropionate concentration in coral reef invertebrates varies according to species assemblages
Source: Sci Rep. 2020 Jun 18;10:9922. doi: 10.1038/s41598-020-66290-5 (PMC7303174; doi:10.1038/s41598-020-66290-5)
Supplement: Supplementary file 1 — Supplementary data S1. [file 41598_2020_66290_MOESM1_ESM.pdf]

Supplementary data S1

Dimethylsulfoniopropionate concentration in coral reef invertebrates varies according to species assemblages.

Isis Guibert, Flavien Bourdreux, Isabelle Bonnard, Xavier Pochon, Vaimiti Dubousquet, Phila Raharivelomanana, Véronique Berteaux-Lecellier and Gael Lecellier.

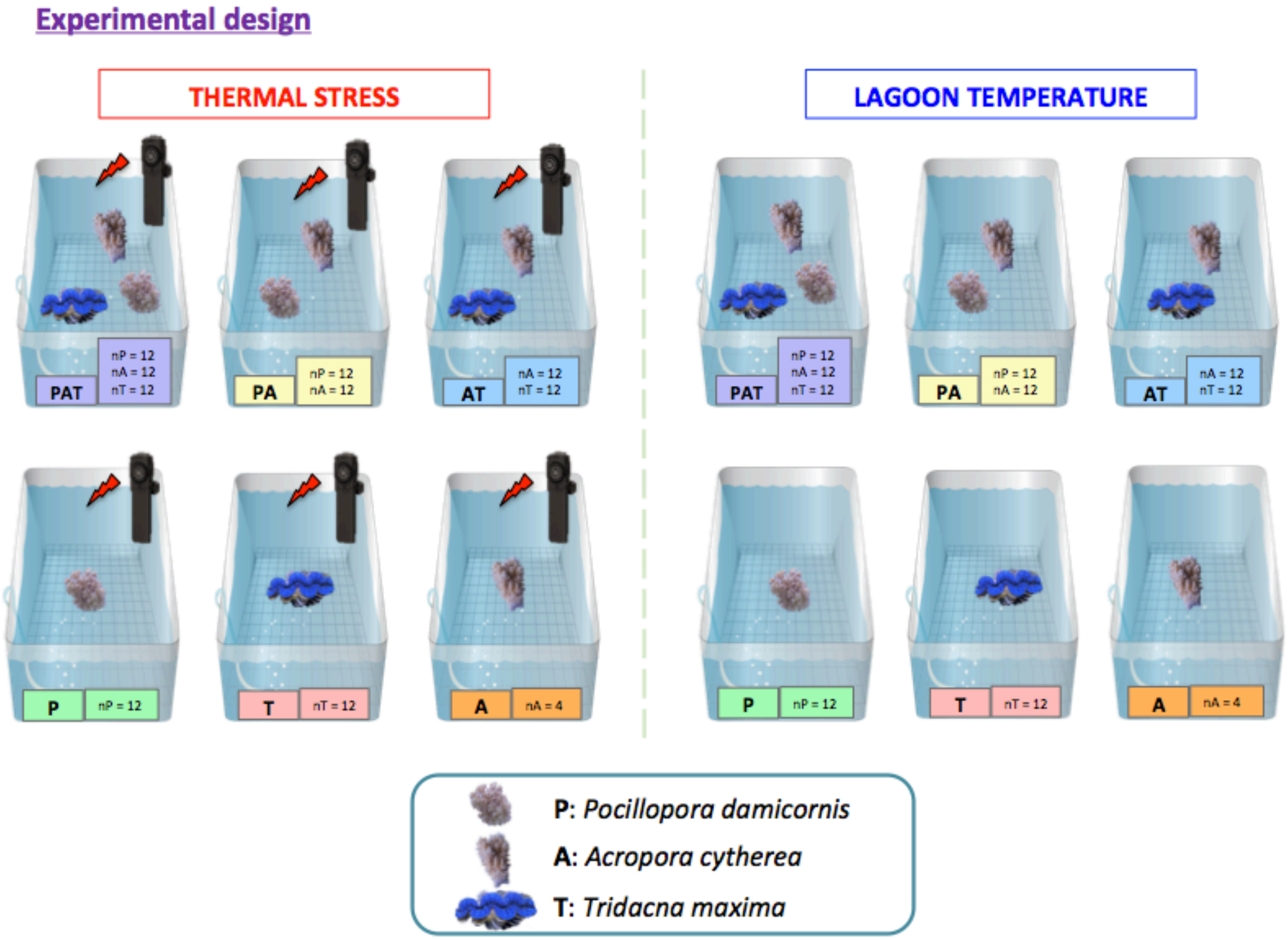

Supplementary 1a: Experimental design. N is the total number of coral nubbins or giant clam per aquarium. The figure was realized by Guibert Isis and adapted from Guibert et al. (2020) with the use of pictures from Guibert Isis, and aquarium tank and airstone drawing from Tracey Saxby, Integration and Application Network, University of Maryland Center for Environmental Science (ian.umces.edu/imagelibrary/).

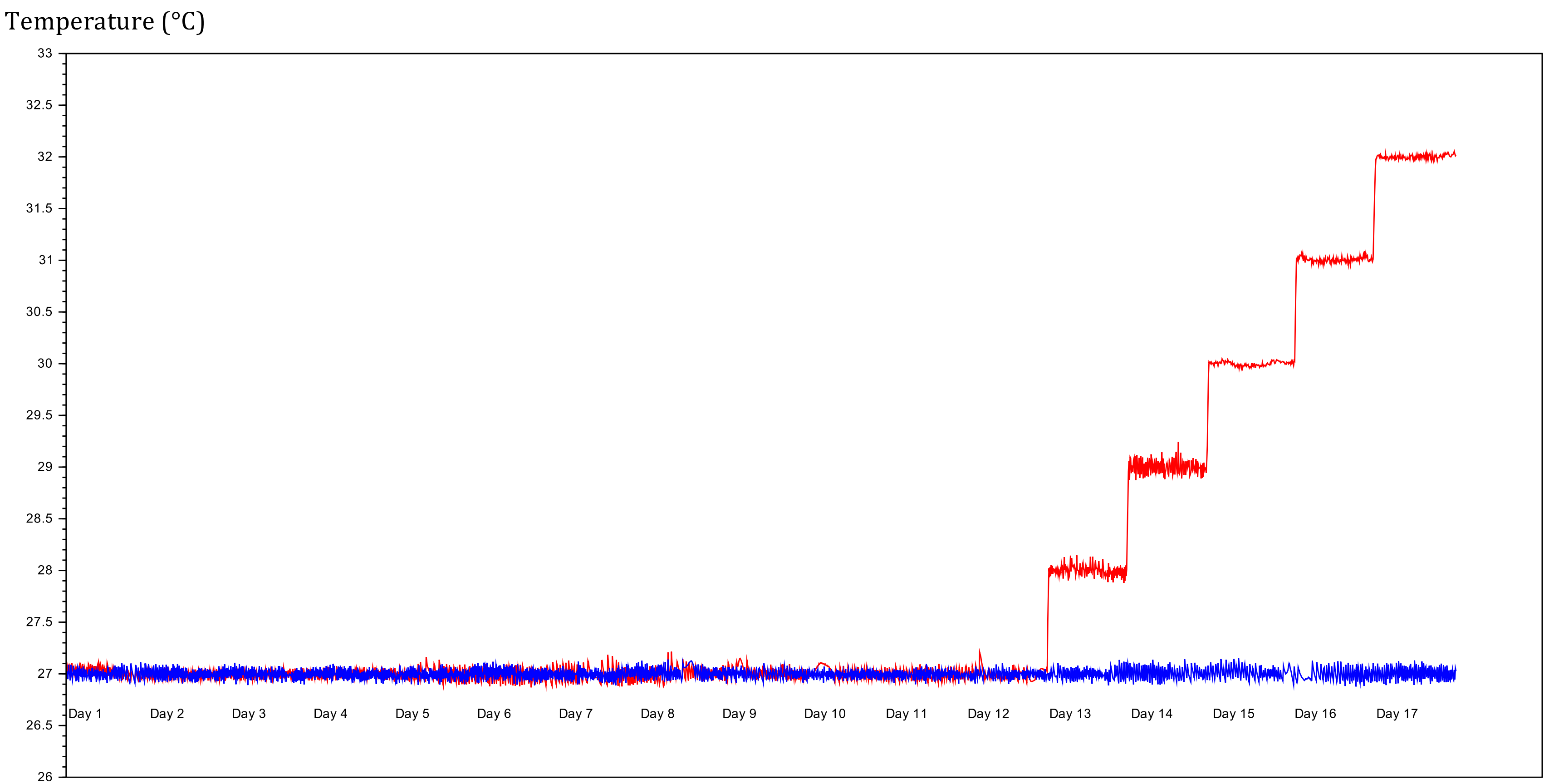

Supplementary 1b: Mean temperature (°C) according to the numbers of days in the aquarium under thermal stress (red) or lagoon temperature (blue).

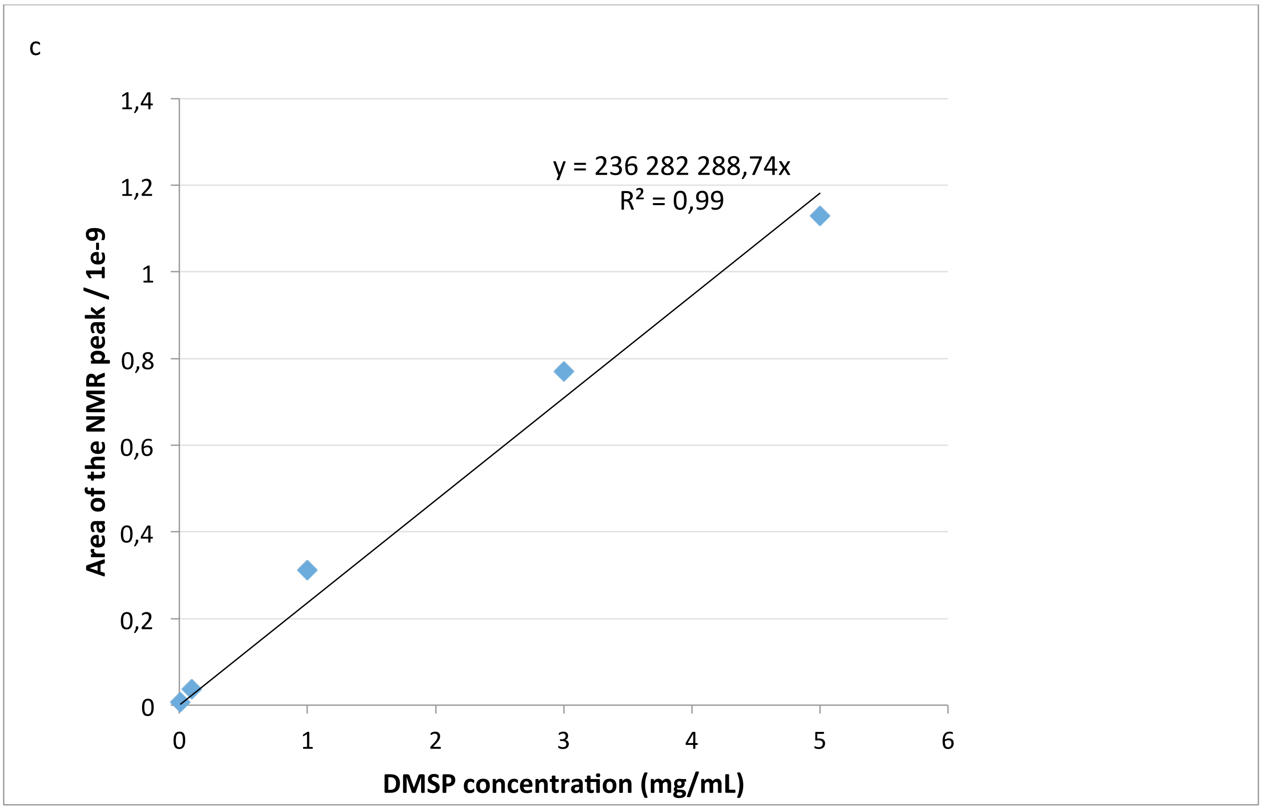

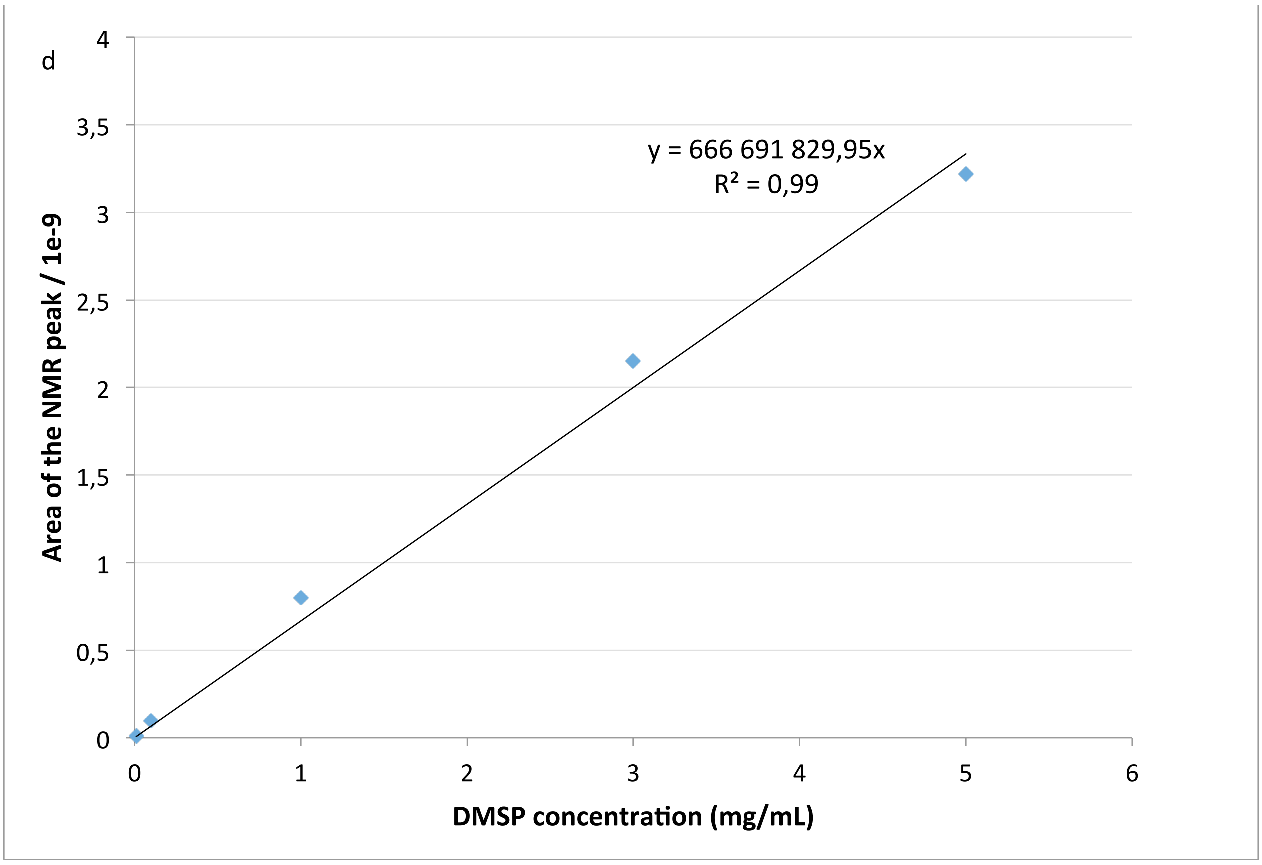

Supplementary data S1c-d: Calibration curve of DMSP concentration for corals (c) or giant clams (d). Areas of the NMR peak were determined by NMR analysis from standards DMSP concentration.

e

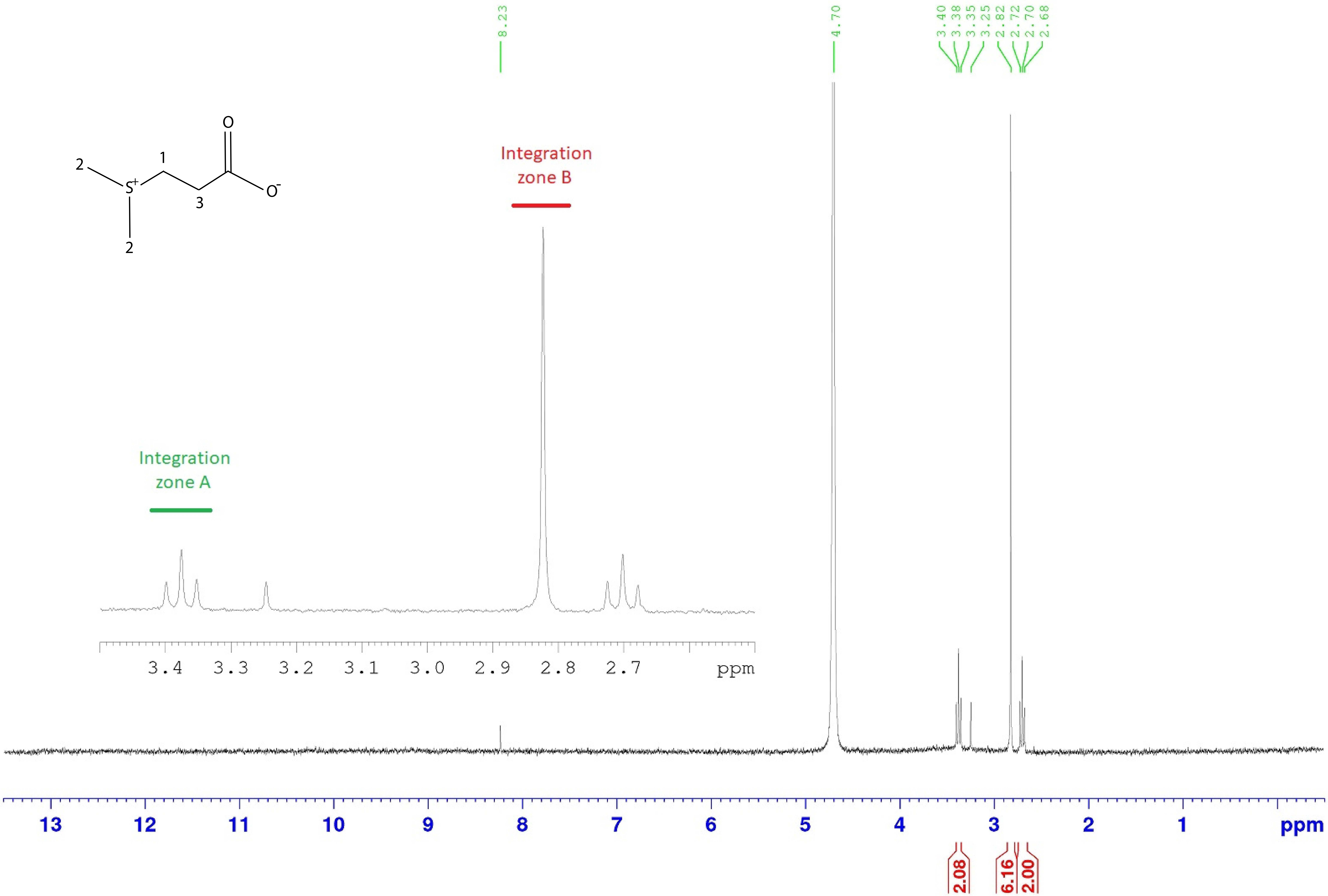

2
1
O
S+
3
O-
2
Integration
zone B
Integration
zone A
8.23
4.70
3.40
3.38
3.35
3.25
2.82
2.72
2.70
2.68
3.4 3.3 3.2 3.1 3.0 2.9 2.8 2.7 ppm
13 12 11 10 9 8 7 6 5 4 3 2 1 ppm
2.08
6.16
2.00

f

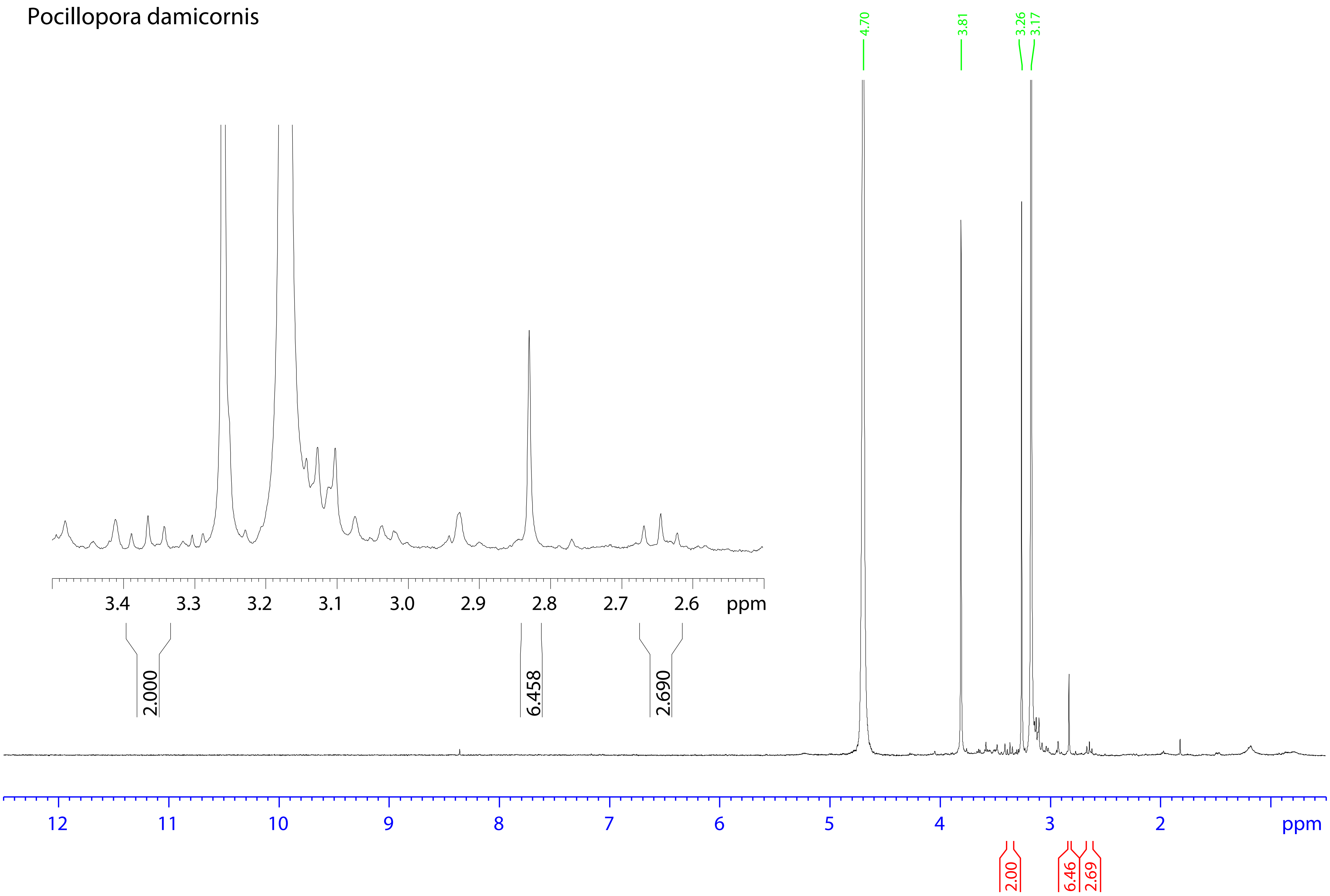
Pocillopora damicornis
4.70
3.81
3.26
3.17
3.4
3.3
3.2
3.1
3.0
2.9
2.8
2.7
2.6
ppm
2.000
6.458
2.690
12
11
10
9
8
7
6
5
4
3
2
ppm
2.00
6.46
2.69

g

Acropora cytherea

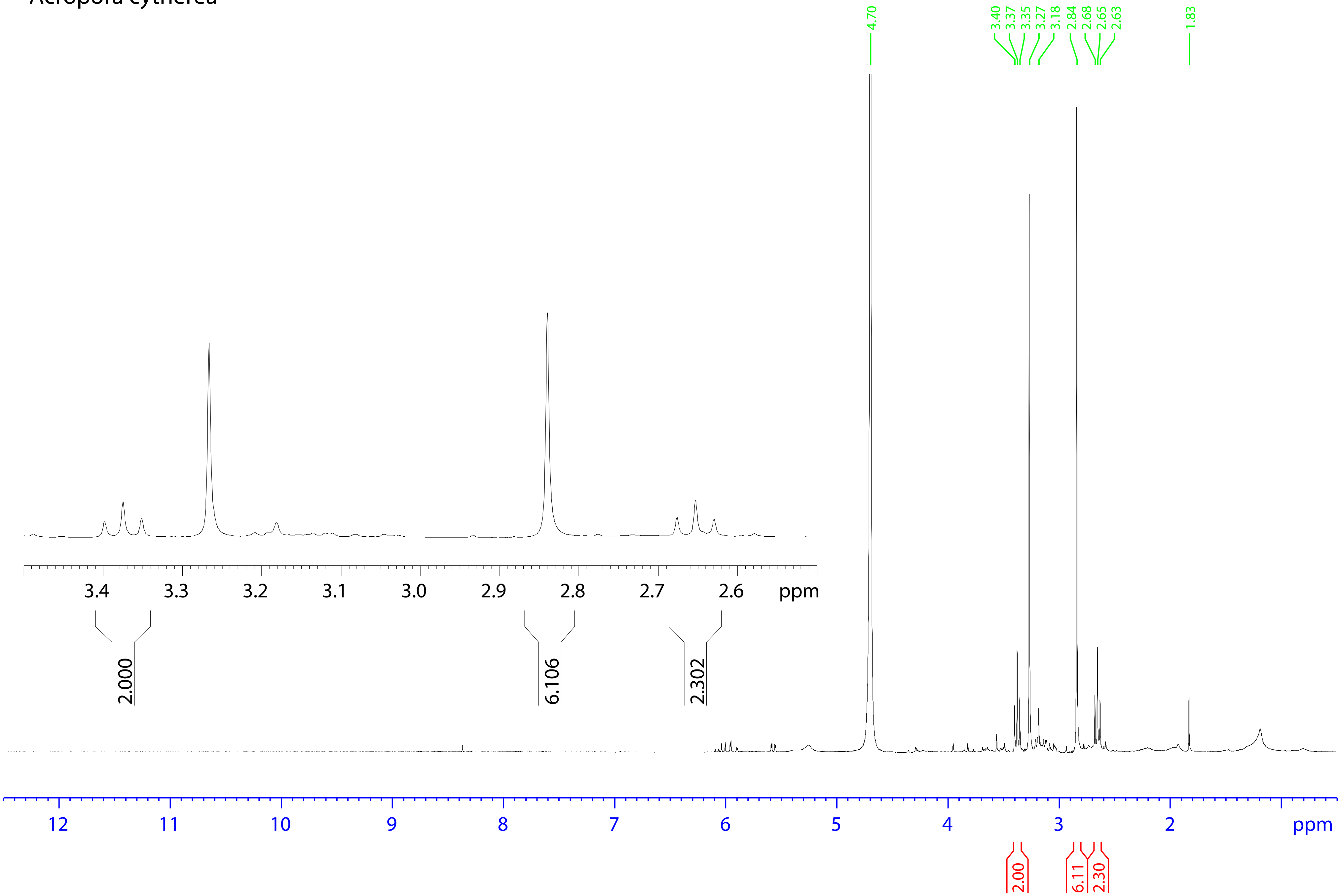

h

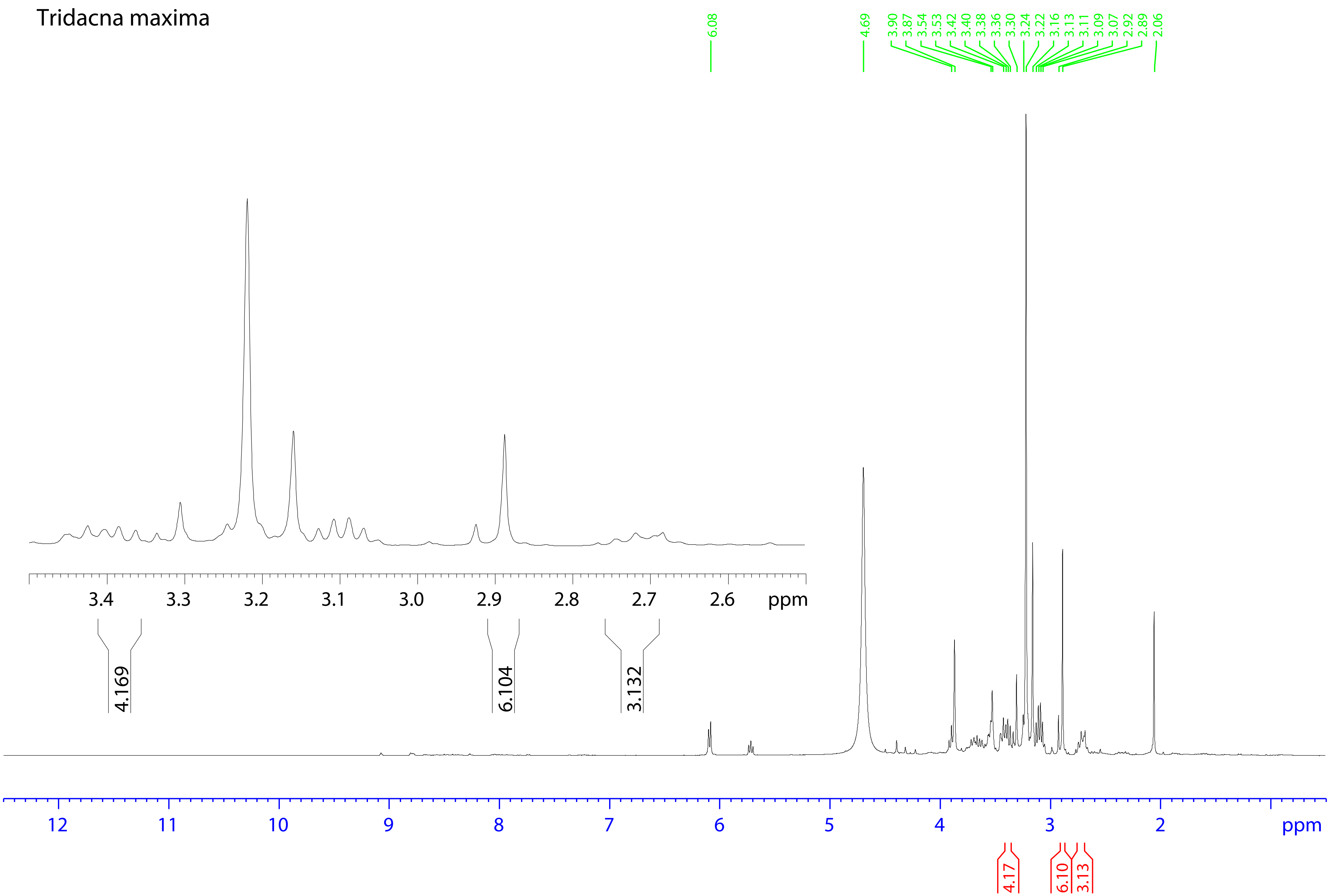

Supplementary data S1e-h. Representation of the Noise region (e), the integration zone A (used for Pocillopora damicornis (f) and Acropora Cytherea (g)) and B (for Tridacna maxima (h)).
